# Spin-stripe phase in a frustrated zigzag spin-1/2 chain

**DOI:** 10.1038/ncomms8255

**Published:** 2015-06-11

**Authors:** M. Pregelj, A. Zorko, O. Zaharko, H. Nojiri, H. Berger, L. C. Chapon, D. Arčon

**Affiliations:** 1 Jožef Stefan Institute, Jamova c. 39, Ljubljana 1000, Slovenia; 2 Laboratory for Neutron Scattering and Imaging, Paul Scherrer Institute, Villigen CH-5232, Switzerland; 3 Institute for Materials Research, Tohoku University, Sendai 980-8577, Japan; 4 Ecole polytechnique fédérale de Lausanne, Lausanne CH-1015, Switzerland; 5 Institut Laue-Langevin, BP 156X, Grenoble 38042, France; 6 Faculty of Mathematics and Physics, University of Ljubljana, Jadranska c. 19, Ljubljana 1000, Slovenia

## Abstract

Motifs of periodic modulations are encountered in a variety of natural systems, where at least two rival states are present. In strongly correlated electron systems, such behaviour has typically been associated with competition between short- and long-range interactions, for example, between exchange and dipole–dipole interactions in the case of ferromagnetic thin films. Here we show that spin-stripe textures may develop also in antiferromagnets, where long-range dipole–dipole magnetic interactions are absent. A comprehensive analysis of magnetic susceptibility, high-field magnetization, specific heat and neutron diffraction measurements unveils *β*-TeVO_4_ as a nearly perfect realization of a frustrated (zigzag) ferromagnetic spin-1/2 chain. Notably, a narrow spin-stripe phase develops at elevated magnetic fields due to weak frustrated short-range interchain exchange interactions, possibly assisted by the symmetry-allowed electric polarization. This concept provides an alternative route for the stripe formation in strongly correlated electron systems and may help understanding of other widespread, yet still elusive, stripe-related phenomena.

## Abstract (web summary)

Stripe phases in nature often form due to the competition between long- and short-range interactions. Here, Pregelj *et al*. demonstrate the emergence of a spin-stripe phase in the absence of long-range magnetic interactions in a frustrated spin chain material *β*-TeVO_4_.

## Graphical abstract

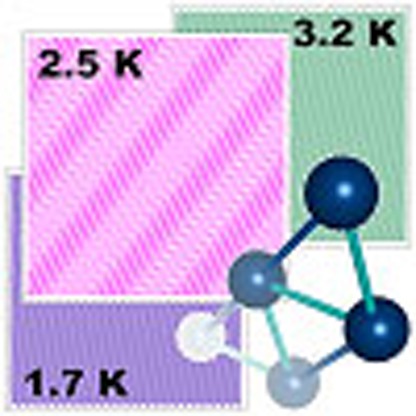

Stripe patterns appear in remarkably diverse materials, ranging from biological to strongly correlated electron systems^1^,^2^,^3^,^4^,^5^,^6^,^7^,^8^,^9^,^10^,^11^,^12^,^13^,^14^,^15^. The common driving force behind stripe formation is a natural tendency of a system to balance two or more rival phases that appear because of competing interactions. For instance, in ferromagnetic thin films, spin-up and spin-down magnetic domains form stripe textures because of opposing short-range exchange and long-range dipole–dipole interactions^4^,^5^,^6^,^7^. In antiferromagnets, however, there is no finite magnetization that could mediate long-range interactions between magnetic domains, so the stripe formation is not expected. Nevertheless, stripe modulation may also develop due to the coupling between different order parameters that is mediated solely by short-range forces, as has been observed in some biological membrane systems^8^,^9^,^10^. Similar mechanisms can also be active in antiferromagnets and may be relevant to other strongly correlated electron systems, for example, to high-temperature superconductors, where the competition between charge-stripe ordering^11^,^12^,^13^ and superconductivity has become increasingly apparent^14^,^15^. Therefore, any evidence of stripe formation in the absence of long-range interactions in strongly correlated electron systems is of utmost importance.

A convenient system to search for this intriguing phenomenon is a frustrated (zigzag) quantum spin-1/2 chain, because of its extremely rich phase diagram. Most importantly, the energy difference between collinear and spiral spin states can be accurately tuned by the applied magnetic field, *B*^16^,^17^,^18^,^19^,^20^. In particular, when the nearest-neighbour (NN) interaction (*J*_1_) is ferromagnetic and the next-nearest-neighbour (NNN) one is antiferromagnetic (*J*_2_>0), that is, in the case of a frustrated ferromagnetic zigzag chain, a vector-chiral (VC) ground state is at elevated magnetic fields succeeded by collinear spin-density-wave (SDW) phases^16^,^17^, with two- (*p*=2) or three-magnon (*p*=3) bound states (Fig. 1a). Moreover, when magnetization *M* approaches saturation (*M*/*M*_sat_=1), intriguing multipolar orders develop^16^,^17^. Although several copper-oxide^21^,^22^ and vanadate^23^,^24^,^25^ compounds possess the ‘zigzag' chain topology, high saturation fields, anisotropy effects or sizable interchain interactions hamper experimental investigation in most cases.

Here we highlight *β*-TeVO_4_ where chains of corner-sharing VO_5_ pyramids run along the *c* axis^26^,^27^. So far, this compound has been treated as a simple V^4+^ spin-1/2 chain system with only the NN antiferromagentic interaction (*J*_1_=21.4(2) K)^27^, as the maximum in the magnetic susceptibility, *χ*(*T*), at 14 K can be described by the Bonner–Fischer model^28^. However, high-temperature susceptibility yields a very small positive (ferromagnetic) Curie–Weiss temperature *θ*, varying from 1.6 to 4.4 K, depending on the magnetic field orientation^27^, which implies the importance of both the NN V–O–V and the NNN V–O–Te–O–V exchange interactions (Fig. 1b) and their opposite sign. Finally, three subsequent magnetic transitions were reported at *T*_N1_=4.65 K, *T*_N2_=3.28 K and *T*_N3_=2.28 K (ref. 27), signifying several energetically almost equivalent magnetic states—a signature of competing magnetic interactions and strong frustration. In this work, we present magnetic susceptibility, magnetization, specific heat, neutron diffraction and spherical neutron polarimetry measurements, which reveal that competing ferromagnetic NN and antiferromagnetic NNN interactions in *β*-TeVO_4_ are comparable in magnitude. The derived magnetic phase diagram corroborates main features of the theoretical one (Fig. 1), thus making *β*-TeVO_4_ a nearly ideal realization of the frustrated ferromagnetic spin-1/2 chain model, where quadrupolar/spin-nematic phase is predicted already at 18.7 T. Moreover, a striking new stripe phase has been discovered between the theoretically predicted VC and SDW phases. In the corresponding magnetic field range, the strength of the interchain interaction matches the energy difference between these two parent phases and thus allows for the establishment of the nanoscale modulation of the magnetic order even in the absence of sizable long-range dipole–dipole magnetic interactions. Possible electric polarization that is in the VC state allowed by symmetry may further assist the stripe formation. This makes *β*-TeVO_4_ an intriguing manifestation of the spin-stripe modulation in an antiferromagnet, where stripe formation appears to be driven by the coupling between order parameters, a mechanism so far almost exclusively attributed to biological systems^8^,^9^,^10^.

## Results

### Signatures of multiple magnetic transitions

In agreement with the published results^27^, our magnetic susceptibility data *χ*(*T*) above 80 K for **B**||*a* yield ferromagnetic character (*θ*=2.7(1) K; see Methods) as well as a clear maximum at much higher temperature of 14(1) K (Fig. 2a). Similarly, specific-heat results show a pronounced hump at 12(1) K (Fig. 2b), which corresponds to the magnetic part of the specific heat *c*_mag_ that develops on top of the usual lattice contribution *c*_latt_ (ref. 29; see Methods), as expected for a low-dimensional system. At lower temperatures, distinct *λ*-type anomalies are observed at *T*_N1_ and *T*_N3_, whereas the kink at *T*_N2_ is more subtle (Fig. 2d). When the magnetic field of 3 T is applied along the *a* axis, the anomaly at *T*_N3_ disappears, implying that the zero-field magnetic ground state is suppressed.

**Figure 1:**
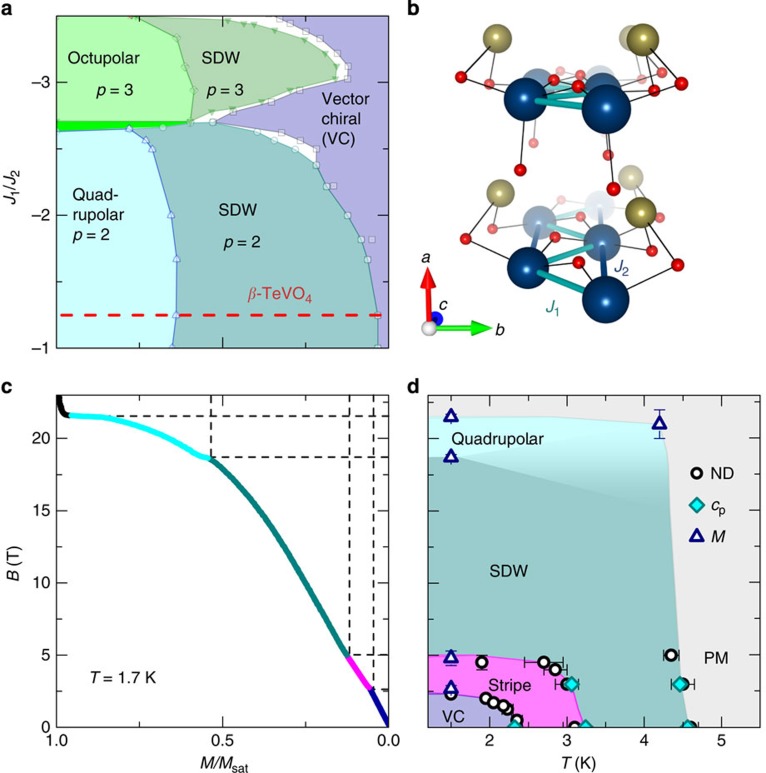
Comparison of the theoretical and experimental phase diagrams. (**a**) Schematic phase diagram of the frustrated ferromagnetic spin-1/2 chain model as a function of *J*_1_/*J*_2_ and *M*/*M*_sat_ (for details see ref. 16), where VC and SDW denote vector-chiral and spin-density-wave phases, respectively, whereas *p* denotes the order of the bound magnon state. The dashed line corresponds to *J*_1_/*J*_2_=−1.25, found in *β*-TeVO_4_. (**b**) The crystal structure of *β*-TeVO_4_, with zigzag-chain interactions. Small, medium and large spheres denote O^2−^, Te^4+^ and the magnetic V^4+^ ions, respectively. (**c**) Magnetization normalized to the saturation value (*M*/*M*_sat_) measured in the magnetic field along the *a* axis and (**d**) the experimental magnetic phase diagram of *β*-TeVO_4_. PM indicates the paramagnetic state, whereas ND, *c*_p_ and *M* denote data points derived from neutron diffraction, specific heat and magnetization measurements, respectively. Error bars denote the experimental uncertainty of the magnetic transitions.

**Figure 2:**
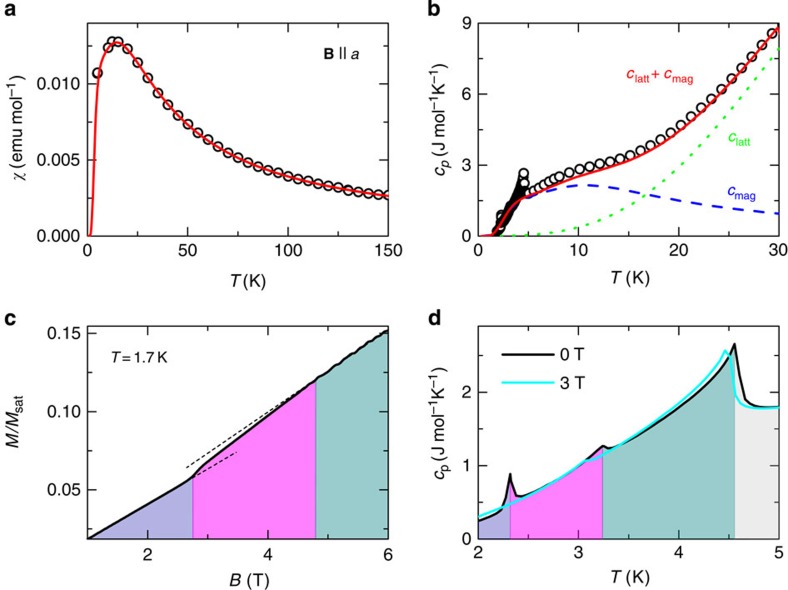
Bulk measurements. (**a**) Magnetic susceptibility (symbols) at 0.1 T (**B**||*a*) and the fit to the *J*_1_/*J*_2_ zigzag chain model considering an average 
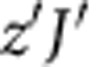
 interchain coupling in the mean-field approximation (line). (**b**) Specific heat in zero-field (symbols) and the fit (*c*_latt_+*c*_mag_; solid line), considering the lattice (*c*_latt_; dotted) and the magnetic (*c*_mag_; dashed) contributions. (**c**) The low-field part of the magnetization for **B**||*a*. The dashed lines are linear extrapolations, indicating two magnetic transitions. (**d**) The low-temperature specific heat measured at 0 and 3 T (**B**||*a*). The colour coding denotes the VC (purple), stripe (magenta) and SDW (dark cyan) ordered phases and the disordered paramagnetic phase (grey).

This corroborates with the magnetization measured for the same orientation of the magnetic field at 1.7 K, that is, well below *T*_N3_, which exhibits two distinct anomalies at *B*_c1_=2.8 T (Fig. 2c) and at *B*_c3_=18.7 T and finally reaches full saturation at *B*_sat_=21.5 T (Fig. 1c). A detailed inspection reveals an additional inflection point at *B*_c2_=4.5(4) T (Fig. 2c). Evidently, below *T*_N3_, *β*-TeVO_4_ undergoes three magnetic-field-induced transitions before reaching full magnetization saturation.

### Incommensurability revealed by neutron diffraction

**Figure 3:**
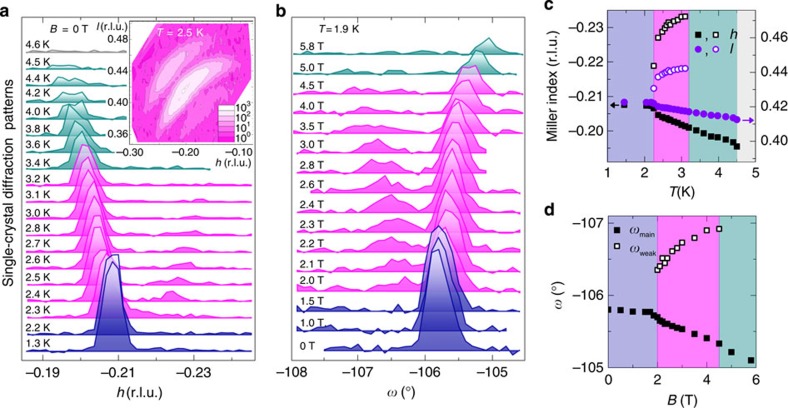
Neutron diffraction results. (**a**) The temperature evolution of the main (−0.208 0 0.423) magnetic reflection. Inset: the corresponding **k**-map measured at 2.5 K. (**b**) The magnetic-field dependence (**B**||*a*) of the (0.208 2 0.577) reflection at 1.9 K and the derived (**c**) temperature- and (**d**) field-dependent position of the main (solid symbols) and weak (open symbols) magnetic reflections. The colour coding denotes the VC (purple), stripe (magenta) and SDW (dark cyan) ordered phases and the disordered paramagnetic phase (grey).

Below *T*_N3_ the neutron experiments disclose a zero-field spiral magnetic ground state that can be described with a single magnetic wave vector **k**=(−0.208 0 0.423) (Fig. 3a; see Methods). The observed incommensurability is a clear signature of the magnetic frustration. Therefore, the modelling of *χ*(*T*) with the Bonner–Fisher model of the simple NN spin-1/2 chain^27^ is not appropriate. Considering the zigzag-chain structure (Fig. 1b), the frustration in *β*-TeVO_4_ must originate from the competition between the NN and the NNN exchange interactions, *J*_1_ and *J*_2_, respectively. Indeed, the NN bond is similar to the corner-sharing V–O–V bridge in CdVO_3_ (ref. 30) and may carry ferromagnetic exchange^31^. The NNN bridge, on the other hand, involves the Te^4+^ lone-pair cation, capable of bridging sizable antiferromagnetic exchange interactions^32^. The pitch angle between two NN spins in a zigzag chain is classically defined as *φ*=arccos(−*J*_1_/4*J*_2_) (refs 33, 34), reflecting in *k*=*φ*/2*π*. However, as the two NN V^4+^ ions in *β*-TeVO_4_ lie in the same unit cell, that is, *k*_*z*_ associates NNN spins, the experimentally determined *k*_*z*_=0.423 must be scaled by 1/2, which yields the NN pitch angle *φ*_c_=0.423 *π* and thus |*J*_1_/*J*_2_|∼1. Taking into the account the experimentally determined Curie–Weiss temperature *θ*=2.7(1) K (Methods), which is even smaller than the ordering temperature *T*_N1_=4.65 K, the competing *J*_1_ and *J*_2_ interactions must have different signs (*J*_1_/*J*_2_∼−1). In fact, to assure the competition *J*_1_ must be ferromagnetic and *J*_2_ antiferromagnetic. In contrast to the chain (*c*) direction, the two neighbouring spins along the *a* axis lie in the neighbouring unit cells. The experimentally derived *k*_*z*_/*k*_*x*_∼−2 thus yields a similar pitch angle along the *a* axis, *φ*_a_∼−*φ*_c_. The V–O interchain distance (*d*=2.81(5) Å, see Methods) is much larger than the typical V–O exchange leg in vanadates, ∼2.0(1) Å (refs 35, 36, 37), hence, the interchain exchange most likely proceeds via Te^4+^ ions, as frequently found in oxyhalide tellurites^32^. In this respect, two very similar interchain V–O–Te–O–V exchange paths exist (see Methods), suggesting that the interchain interactions are also frustrated and that the exact relation between *φ*_a_ and *φ*_c_ is nontrivial.

### Quantitative determination of the exchange interactions

To place our system in the general zigzag spin-chain phase diagram (Fig. 1a)^16^,^17^,^18^, we still need to determine the magnitude of the exchange interactions. This can be determined from modelling of *χ*(*T*). We fit *χ*(*T*) using the exact-diagonalization results for the isolated zigzag chain *χ*_zig_(*T*) (refs 24, 38, 39) with an additional interchain exchange within a mean-field approach^24^,^40^, *χ*_coupled_(*T*)=*χ*_zig_/(1–*λχ*_zig_). Here 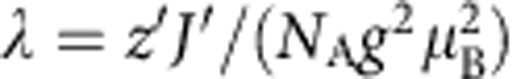, 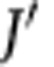 is the interchain coupling to 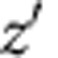 spins on the neighbouring chains, *N*_A_ is the Avogadro constant, *μ*_B_ is the Bohr magneton and *g*=2.01(1) is the gyromagnetic factor for V^4+^ (see Methods). An excellent agreement with the experiment is obtained for *J*_1_/*J*_2_=−1.25, with *J*_1_=38.3 K and 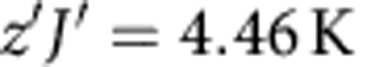 (Fig. 2a), which is in good agreement with the classical estimate *J*_1_/*J*_2_∼−1 derived from the experimentally determined *k*_*z*_. We note that the increased |*J*_1_/*J*_2_| value is an expected effect of quantum fluctuations for spin-1/2 systems^33^. On the other hand, fitting with *J*_1_/*J*_2_>0 yields unrealistically large 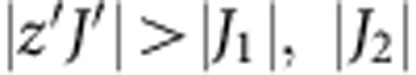. Moreover, the calculated Curie–Weiss temperature^41^ 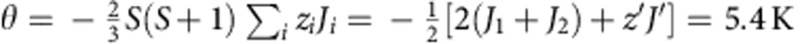 is in fair agreement with the experimental value *θ*=2.7(1)  K, considering an order of magnitude larger exchange interactions. Finally, using *c*_mag_(*T*) 38,39 for the same *J*_1_, *J*_2_ and adding it to the lattice contribution *c*_latt_(*T*) within the Debye model (see Methods), we obtain an excellent fit of the specific heat data (Fig. 2b), thus affirming the extracted exchange parameters.

### Determination of the magnetic phase diagram

To explore all magnetic phases of *β*-TeVO_4_, we performed detailed temperature- and magnetic field-dependent neutron diffraction experiments, focusing on the strongest magnetic reflection (Fig. 3). In zero field, the reflection emerges at *T*_N1_=4.5(1) K at **k**=(−0.195 0 0.413) and shifts to larger |*h*| and |*l*| with decreasing temperature. At *T*_N2_=3.1(1) K, the main **k** reflection is accompanied by an additional weak reflection appearing at **k′**=(−0.233 0 0.442). On further cooling, this reflection shifts towards the main one and at *T*_N3_=2.20(3) K, they finally collapse into a single peak at **k**=(−0.208 0 0.423). This peak does not shift with temperature at least down to 1.6 K (Fig. 3c), indicating the stability of the magnetic ground state. Very similar response is observed below *T*_N3_ when magnetic field is applied along the *a* axis. Above *B*_c1_, the weak **k′** reflection separates from the main **k** reflection and eventually disappears at *B*∼*B*_c2_ (Fig. 3b). It thus appears that both external controlling parameters, that is, the temperature and the applied magnetic field, drive the system through the same magnetic phases.

Further details of the three magnetic phases are provided by zero-field spherical neutron polarimetry. In our study (see Methods), we focused on the strongest magnetic reflection (−0.208 0 0.423). In particular, we measured the full polarization matrix at 1.6 K and then followed the temperature evolution of chiral *yx* and non-chiral *yy* terms (Table 1). Measurements in the (*h*00)/(00*l*) scattering plane reveal a significant chiral term below *T*_N3_, indicating an imbalance in the chiral-domain population. Above *T*_N3_, on the contrary, the chiral term is almost completely suppressed, implying that chirality is either dramatically reduced or the plane of the spiral is reoriented along the scattering plane. This complies with the behaviour of the non-chiral term, which above *T*_N3_ significantly increases, indicating a reorientation of the magnetic moments. Measuring the same reflection in the (−*h*02*h*)/(0*k*0) scattering plane allows probing a different projection of the magnetic structure factor. We find that the chiral term is now absent in the entire temperature range (Table 1), whereas the non-chiral term again increases above *T*_N3_. We can thus conclude that (i) the plane of the spiral, existing below *T*_N3_, is very close to the (−*h*02*h*)/(0*k*0) scattering plane, (ii) at *T*_N3_ the magnetic moments reorient and (iii) above *T*_N3_ the magnetic order remains incommensurate, but is dominated by collinear amplitude modulation (SDW).

All our data are summarized in the magnetic phase diagram (Fig. 1d), which we compare to the theoretical diagram for the frustrated ferromagnetic (*J*_1_/*J*_2_<0) zigzag spin-1/2 chain model in Fig. 1. This comparison is best presented when the low-temperature magnetization data (Fig. 1c) are used for scaling between *B* and *M*/*M*_sat_. The zero-field spiral magnetic ground state clearly confirms the predicted VC state. Above *B*_c2_, the intensity of the magnetic reflection significantly changes (Fig. 3b), whereas the magnetic order remains incommensurate. Hence, the remarkable agreement between the theoretical and experimental *M*/*M*_sat_ values at *B*_c2_ and *B*_c3_ (Fig. 1) suggests that the VC phase (*B*<*B*_c1_) is followed by the SDW phase (*B*_c2_<*B*<*B*_c3_). This complies with spherical neutron polarimetry results, indicating the presence of SDW above *T*_N3_. The SDW phase is according to the model succeeded by the quadrupolar/spin-nematic phase persisting up to the saturation (*B*_c3_<*B*<*B*_sat_). We note that the steep magnetization slope in this region differs from the gentle slope experimentally observed in LiCuVO_4_ (ref. 23), presumably due to the different interchain couplings in the two systems. However, the narrow phase between *B*_c1_ and *B*_c2_, where the weak **k′** reflection complements the main **k** magnetic reflection, is observed for the first time.

### Emergence of a novel spin-stripe phase

**Figure 4:**
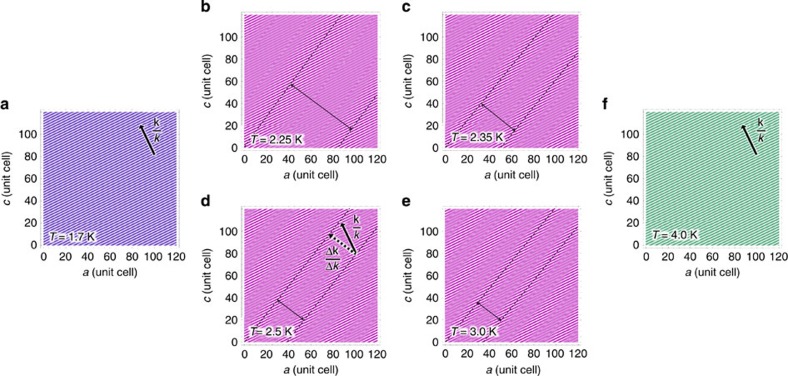
Temperature evolution of the magnetic structure modulation. The zero-field magnetic structure modulation (**a**) in the VC phase at 1.7 K, (**b**–**e**) in the stripe phase between 2.25 and 3.0 K, where super-satellite reflections reveal additional nanometre-scale modulation, and (**f**) in the SDW phase at 4.0 K, where super-satellite reflections are absent again. Different magnetic phases, that is, the VC (purple), stripe (magenta) and SDW (dark cyan) phases, are indicated by different colours. The colour scale denotes the magnetic modulation, the direction of which, **k**/*k*, is indicated by thick arrows. In the stripe phase, the direction of the additional stripe modulation, Δ**k**/Δ*k*, is indicated by the thick dotted arrow. The thin dashed lines emphasize the centres of the stripes, allowing for the estimate of the stripe modulation period (two sided arrows), which amounts to (**b**) 30.7, (**c**) 17.8, (**d**) 12.7 and (**e**) 12.3 nm.

To gain more information about the peculiar **k′** reflection, we measured an extended **k**-map at 2.5 K, that is, between *T*_N2_ and *T*_N3_ (inset in Fig. 3a). Evidently, the main magnetic reflection at **k**(2.5 K)=(−0.203 0 0.419) is accompanied with two ‘super-satellite' reflections symmetrically shifted by ±Δ**k**(2.5 K)=±(−0.030 0 0.021) from **k** (2.5 K). This indicates that the incommensurate magnetic structure in this phase experiences an additional nanometre-scale stripe modulation with well-defined modulation period of 12.7 nm, that is, ∼40 lattice units (Fig. 4d). Moreover, by heating from 2.25 to 3.1 K, this modulation period can be changed between 30 and 12 nm, respectively (Fig. 4b–e). We stress that the width of all magnetic reflections, that is, the main as well as satellite ones, is resolution limited, indicating that both magnetic modulations (**k** and Δ**k**) are very well defined, which is a clear evidence of a coherent and uniform magnetic state rather than a simple coexistence of the VC and the SDW phases. The observed Δ**k** modulation markedly differs from typical solition lattices, which develop in the insulating SDW phases at low temperatures. There the sinusoidal modulation squares up, reflecting in 3**k**, 5**k** and higher-order harmonic reflections^42^,^43^,^44^. In fact, the observed behaviour is rather reminiscent of domain patterns observed in two-component systems^1^.

## Discussion

To thoroughly address the novel stripe phase, we first need to understand the two adjacent phases. The zero-field VC ground state is defined by **k**, which complies with the spin-1/2 zigzag chain model^16^, yielding *J*_1_/*J*_2_=−1.25. The same model accounts for the collinear SDW phase at higher magnetic fields or temperatures, exhibiting a weak field and temperature dependence of the main magnetic reflection, **k**(*T*, *B*). On the contrary, the nanometre-scale modulation in the stripe phase is far from being parallel to the main **k** modulation (Fig. 4b–e), that is, Δ**k**·**k**≠0, hence, the stripes cannot be explained by the intrachain interactions alone.

The role of the weak interchain interactions 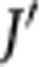 (see Methods) in the stripe phase must be related to the competition between the VC and SDW phases. Namely, with increasing magnetic field, the energy difference between these phases decreases and at *B*∼*B*_c1_ it becomes comparable to the strength of 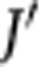. Similarly, the increasing temperature leads to the same kind of phase competition near *T*_N3_; its effect on the energy difference between the two states reflects in the temperature-dependent **k**(*T*) (Fig. 3d). In the case of non-frustrated 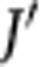, |*k*_*z*_/*k*_*x*_|≡2 would be independent of the magnetic field or temperature. This is contradicted by the experiment, as the ratio |*k*_*z*_/*k*_*x*_| noticeably changes from 2.11 to 2.04 between *T*_N1_ and *T*_N3_, respectively (Fig. 3). Therefore, 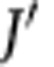 must be frustrated and is thus most probably essential for the nanometre-scale stripe modulation (Fig. 4b–e) that releases the degeneracy of the VC and SDW states.

Moreover, the observed stripes are in phase, as the satellite reflections appearing around a sharp central magnetic peak are also sharp (inset to Fig. 3a). Other presently known spin stripe systems, for example, ferromagnetic stripe domains 7 and spin stripes in high-temperature superconductors^13^, have antiphase domain boundaries, which reflect long-range repulsive forces. This difference implies that a conceptually different coupling mechanism must be responsible for the stripe formation in *β*-TeVO_4_.

Indeed, the observed stripe state is antiferromagnetic and therefore lacks magnetized domains that could mediate sizable long-range magnetic (dipole–dipole) interactions. As the stripe phase is sandwiched between the VC and SDW phase, the microscopic mechanism for the stripe formation is likely the coupling of two corresponding order parameters, in the analogy to biological-membrane systems^8^,^9^,^10^. In this case, the stripe formation would rely on the competition between the magnetic entropy contribution that decreases with decreasing temperature and thus favours fully developed moments of the VC phase, and the magnetic anisotropy energy, which in an uniaxial case favours collinear magnetic moments of the SDW phase. This seems to be in line also with finite, although very weak, chiral terms in the intermediate phase (Table 1) and may also lead to asymmetric intensity of satellite reflections (inset in Fig. 3a). The stripe formation may be further assisted by possible electric polarization, as the latter is allowed in the VC phase due to symmetry reasons as argued in magnetoelectric multiferroics^45^. Such a novel state may be inherent to quasi one-dimensional chiral multiferroics, as stripes of spiral and collinear phases have recently been found also in MnWO_4_ (ref. 46).

Our results exceed the limits of standard spin-stripe models assuming that stripe patterns stem from the competition between short-range attractive and long-range repulsive magnetic forces^4^,^5^,^6^,^7^. We demonstrate that *β*-TeVO_4_, which has proven to be a nearly perfect realization of the highly frustrated ferromagnetic spin-1/2 chain, is a model system for spin-stripe formation in the absence of sizable long-range magnetic interactions. The dominant intrachain couplings are accompanied by weak frustrated short-range interchain exchange interactions and possibly also by the electric polarization, which stabilize a nanometre-scale modulation of a purely antiferromagnetic order. The proposed concept thus reveals a new perspective on the stripe pattern formation in strongly correlated electron systems and may prove valuable for understanding of other, yet unexplained, phenomena, for example, the charge modulation in high-temperature superconductors and its relation to superconductivity. Moreover, the frustrated nature of 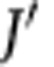 makes this system highly susceptible to external perturbations and, therefore, allows for a direct manipulation of the nanometre-scale modulation by either changing the temperature or the strength of the applied magnetic (and possibly also electric) field. Finally, understanding the origin of the stripe phase may prove essential in the search for the elusive spin-nematic phase that is predicted close to the saturation field, which is in *β*-TeVO_4_ conveniently low in respect to other candidate systems^23^.

*Note added in proof*: After having submitted the manuscript we became aware of a work by Saúl and Radtke^47^ which theoretically investigates the magnetic interactions in the β-TeVO_4_ compound.

## Methods

### Sample description

All measurements were conducted on the same high-quality single-crystal samples. These were obtained from TeO_2_ and VO_2_ powders by chemical vapour transport reaction, using two-zone furnace and TeCl_4_ as a transport agent. Detailed reaction conditions are reported in ref. 48.

### Neutron diffraction

Neutron experiments on a 2 × 3 × 4 mm^3^ single crystal were performed on TriCS diffractometer at the Paul Scherrer Institute (PSI), Switzerland. The zero-field diffraction data between 1.7 and 5 K were collected using a cooling machine in a four-circle geometry. Magnetic field dependences were measured in a vertical magnet in the normal beam (tilt) geometry.

The monoclinic unit cell (space group *P*2_1_/*c*) with parameters *a*=4.3919(1) Å, *b*=13.5155(1) Å, *c*=5.4632(1) Å and *β*=90.779(1)° was determined from single-crystal neutron diffraction data (440 reflections) collected at 10 K. These data were used also to refine the atomic positions (Table 2) and yield the reliability factor *R*_obs_=9.03. The refinement was performed using JANA2006 (ref. 49).

We note that width of nuclear and magnetic peaks measured in transversal to **k** (omega-scans) is comparable and is very close to the instrumental resolution. The **k**-scan presented in the inset in Fig. 3a was performed in a four-circle geometry. The peak shape is dominated by instrumental resolution (with vertical component rather relaxed) and is not intrinsic to the crystal.

The scan in Fig. 3b extended from (0.204 2.05 0.558) at *ω*=−107.5° to (0.211 1.965 0.598) at *ω*=−104.5°.

### Spherical neutron polarimetry

Experiments on a 2 × 3 × 4 mm^3^ single crystal were performed on D3 diffractometer equipped with CRYOPAD operating with wavelength *λ*=0.825 Å at the Institute Laue Langevin (ILL), Grenoble, France. The low-temperature diffraction data between 1.7 and 5 K were collected using ILL Orange cryostat. The two different scattering planes, namely (*h*00)/(00*l*) and (−*h*02*h*)/(0*k*0), were accessed by using two different mountings of the crystal.

### Characterization of the exchange pathways

Derived atomic positions provide an additional insight into the nature of the dominant exchange interactions. In particular, the ferromagnetic nature of *J*_1_ complies with the dependence of the exchange coupling on the distance between the V^4+^ ion and the basal oxygen plane of the VO_5_ square pyramid, *δ*. Namely, for small *δ*, the exchange is ferromagnetic because of the *π*-interaction between vanadium 3*d*_*xy*_ and the basal oxygen 2*p* orbitals, as for instance also found in CdVO_3_ where *δ*=0.430 Å (ref. 31). In *β*-TeVO_4_, we find reasonably small *δ*=0.58(5) Å at 10 K.

On the contrary, the antiferromagnetic character of *J*_2_ and 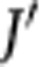 is in agreement with the findings for O–Te–O exchange bridges in oxyhalide tellurites^32^. The derived 
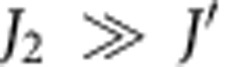
 reflects substantially different lengths of intra- and inter-chain O–Te bonds that equal 1.940(12) Å and 2.939(19) Å, respectively. Finally, assuming that the interchain 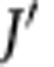 exchange interactions pass through the shortest O–Te interchain distance *d*_Te–O_=2.939(19) Å, we find two similar V–O–Te–O–V paths, involving comparable intrachain O–Te and O–V distances (Table 3).

### Magnetic susceptibility

The magnetic susceptibility *χ*(*T*) for the magnetic field **B** applied along the *a* axis was measured between 2 and 300 K on a Quantum Design physical property measurement system.

**Figure 5:**
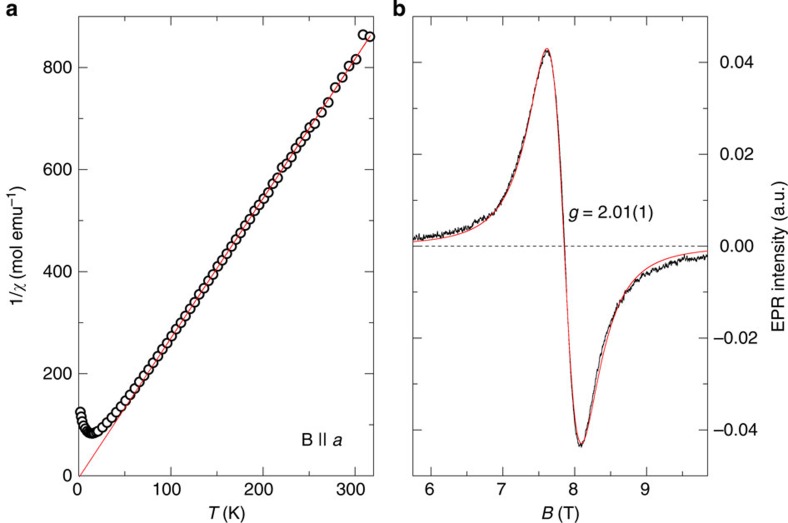
Characterization of the paramagnetic species. (**a**) The inverse of the magnetic susceptibility in the magnetic field of 0.1 T along the *a* axis as a function of temperature (symbols). The solid line represents a linear fit for *T*>80 K. (**b**) Room temperature electron paramagnetic resonance spectrum at 220.8 GHz (black line), and the corresponding fit to the Lorentzian lineshape (red line), yielding the *g* factor of V^4+^ in *β*-TeVO_4_.

The Curie–Weiss temperature *θ* was determined from the linear fit of the inverse susceptibility, 1/*χ*, above 80 K (Fig. 5a), measured in the magnetic field of 0.1 T. The temperature-independent diamagnetic contribution of the core shells is of the order of 10^−5^ emu mol^−1^ and is thus negligible when compared with other terms.

### High-field magnetization measurements

Magnetization measurements in pulsed magnetic fields up to 25 T were performed at the High Magnetic Field Laboratory, Institute for Materials Research, Sendai, Japan.

### Specific heat

The specific-heat measurements were performed at 0 and 3 T between 1.8 and 30 K using the physical property measurement system. The crystal-lattice contribution to the specific heat was modelled with the Debye approximation^29^


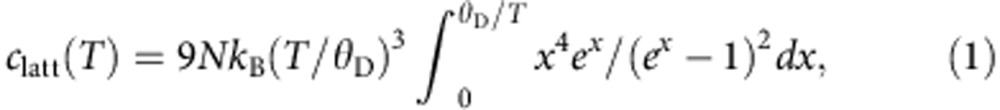


where *θ*_D_ is the Debye temperature, *N* is the number of atoms in the crystal and *k*_B_ is the Boltzmann constant. The best agreement with the experiment was obtained for *θ*_D_=190 K.

### Electron paramagnetic resonance (EPR)

EPR signal was measured at room temperature and 220.8 GHz. The experiment was conducted on a transmission-mode EPR spectrometer at the National High Field Laboratory, Tallhassee, Florida. The line shape is Lorentzian and thus characteristic of exchange narrowing, as evident from the fit shown in Fig. 5b, which yields *g*=2.01(1) and the width Δ*B*=0.830(2) T.

## Additional information

**How to cite this article:** Pregelj, M. *et al*. Spin-stripe phase in a frustrated zigzag spin-1/2 chain. *Nat. Commun.* 6:7255 doi: 10.1038/ncomms8255 (2015).

## Figures and Tables

**Table 1:** Elements of the polarization matrix.

*T* (K)	*Yx*		*yy*	
	S1	S2	S1	S2
1.6	0.33(4)	0.07(5)	0.34(4)	−0.27(5)
2.5	0.11(5)	0.04(7)	1.01(4)	−0.66(7)
3.5	0.04(6)		0.95(4)	

Chiral (*yx*) and non-chiral (*yy*) terms of the polarization matrix of the (−0.208 0 0.423) magnetic reflection, measured in two different scattering planes; S1 (*h*00)/(00*l*) and S2 (−*h*02*h*)/(0*k*0) and corrected for the incomplete polarization of incoming neutrons.

**Table 2:** Fractional atomic coordinates.

Atom	*x*	*y*	*z*
Te	0.0417(9)	0.3910(1)	0.6405(8)
V	0.666(11)	0.158(3)	0.639(11)
O1	0.3047(9)	0.1646(2)	0.6683(9)
O2	0.8294(9)	0.0473(2)	0.8664(9)
O3	0.8062(9)	0.2221(2)	0.9806(9)
O4	0.7467(9)	0.0812(2)	0.3700(9)

The values derived for *β*-TeVO_4_ at 10 K.

**Table 3:** Interatomic distances.

Path	*d*_V–O_	*d*_O–Te_	*d*_Te–O_	*d*_O–V_
1	1.60(5)	2.939(19)	1.940(12)	2.06(5)
2	1.60(5)	2.939(19)	1.854(12)	1.83(5)

The values are given in Å for *β*-TeVO_4_ at 10 K for the most probable interchain exchange interactions (**1**: V-O1-Te-O2-V and **2**: V-O1-Te-O4-V).
